# The Human EST Ontology Explorer: a tissue-oriented visualization system for ontologies distribution in human EST collections

**DOI:** 10.1186/1471-2105-10-S12-S2

**Published:** 2009-10-15

**Authors:** Ivan Merelli, Andrea Caprera, Alessandra Stella, Marcello Del Corvo, Luciano Milanesi, Barbara Lazzari

**Affiliations:** 1 Istituto Tecnologie Biomediche – Consiglio Nazionale delle Ricerche, Via F.lli Cervi 93, Segrate (MI), 20090, Italy; 2 Parco Tecnologico Padano, Via Einstein – Località Cascina Codazza, 26900 Lodi, Italy

## Abstract

**Background:**

The NCBI dbEST currently contains more than eight million human Expressed Sequenced Tags (ESTs). This wide collection represents an important source of information for gene expression studies, provided it can be inspected according to biologically relevant criteria. EST data can be browsed using different dedicated web resources, which allow to investigate library specific gene expression levels and to make comparisons among libraries, highlighting significant differences in gene expression. Nonetheless, no tool is available to examine distributions of quantitative EST collections in Gene Ontology (GO) categories, nor to retrieve information concerning library-dependent EST involvement in metabolic pathways. In this work we present the Human EST Ontology Explorer (HEOE) , a web facility for comparison of expression levels among libraries from several healthy and diseased tissues.

**Results:**

The HEOE provides library-dependent statistics on the distribution of sequences in the GO Direct Acyclic Graph (DAG) that can be browsed at each GO hierarchical level. The tool is based on large-scale BLAST annotation of EST sequences. Due to the huge number of input sequences, this BLAST analysis was performed with the aid of grid computing technology, which is particularly suitable to address data parallel task. Relying on the achieved annotation, library-specific distributions of ESTs in the GO Graph were inferred. A pathway-based search interface was also implemented, for a quick evaluation of the representation of libraries in metabolic pathways. EST processing steps were integrated in a semi-automatic procedure that relies on Perl scripts and stores results in a MySQL database. A PHP-based web interface offers the possibility to simultaneously visualize, retrieve and compare data from the different libraries. Statistically significant differences in GO categories among user selected libraries can also be computed.

**Conclusion:**

The HEOE provides an alternative and complementary way to inspect EST expression levels with respect to approaches currently offered by other resources. Furthermore, BLAST computation on the whole human EST dataset was a suitable test of grid scalability in the context of large-scale bioinformatics analysis. The HEOE currently comprises sequence analysis from 70 non-normalized libraries, representing a comprehensive overview on healthy and unhealthy tissues. As the analysis procedure can be easily applied to other libraries, the number of represented tissues is intended to increase.

## Background

The advent of microarray technology and other techniques that allow for high-throughput genomic analyses has opened the door to large-scale gene expression studies, allowing the observation of transcriptional variation with respect to variable factors of interest, such as different tissues, health states, phenotypes, or clinical outcomes. Moreover, the large number of public EST sequences that are available are a powerful source of information that can be exploited to further enhance the ability to measure gene expression. The National Center for Biotechnology Information (NCBI) dbEST [1] contains more than 8,000,000 human sequences from almost 8,000 libraries, representing a variety of tissues, treatment outcomes, and pathological states. Expression level comparison in EST collections is well implemented on the NCBI Library Browser [2] and Digital Differential Display (DDD) [3] pages, as well as on the *Homo sapiens *Gene Index (HGI) web site [4], where either library-specific or global EST assemblies are presented, and statistically relevant differences among libraries are retrievable. However, at least to our knowledge, no information is currently given with respect to quantitative EST distribution in GO [5,6] classes for the different libraries. The HEOE [7] is a web-based facility that allows comparison of distribution of ontologies in several human EST libraries, representing healthy and diseased tissues. In the HEOE, EST sequence analysis is based on large-scale annotation, retrieval of UniProt and GO identifiers, and classification of proteins in ontology classes, according to the GO DAG. Hierarchical browsing of GO classes and retrieval of related sequences is allowed via the web interface, where data concerning different user-selected tissues can be simultaneously displayed. Statistics on user selected libraries, highlighting GO classes that display significant differences can also be computed. Furthermore, a search facility was implemented to allow the retrieval of ESTs from different libraries that are associated with selected metabolic pathways.

## Results

### The Human EST Ontology Explorer library collection

The whole human EST database, as downloaded from the NCBI, was parsed according to the sequence library-of-origin and libraries were classified according to the preparation method, number of entries and tissue of origin. In order to limit our analyses to libraries that could be considered representative of the tissues' natural expression states, only non-normalized libraries were taken into account. Whenever possible, libraries that had not undergone any other kind of selection in their preparation procedure were preferred, but in some cases libraries enriched in full-length clones were also included in our selection with the aim to provide an exhaustive representation of human tissues, as well as the possibility to compare healthy and unhealthy states in the same tissue. Most of the HEOE libraries include more than 10,000 sequences, but for the previously stated reasons, some libraries including fewer than 10,000 sequences were also considered. A detailed list of the 70 libraries that are included in the HEOE is given in Additional file 1. Nevertheless, libraries number can vary according to the project's development, as already annotated libraries are available, and can be easily added to the HEOE collection.

### Grid computing technology and BLAST annotation

BLASTx [8] annotation was performed on the entire human EST dataset. ESTs were compared with a custom prepared version of the UniProtKB database [9] that was modified to maximize the retrieval of GO identifiers, because only proteins associated with GO identifiers were retained. Due to the large size of both the databases (5.12 GB of EST sequences and 1.06 GB of UniProtKB-derived entries), and considering that each EST sequence was translated on six frames, the computational effort required to complete the analysis was extremely significant. Therefore, we decided to use a grid computing approach to accomplish this task, with the dual intent of speeding up the computation and of providing a benchmark for the scalability on grid of an important bioinformatics procedure such as BLAST.

In detail, we employed the grid infrastructure of the Enabling Grid for E-SciencE (EGEE) project, which relies on the gLite middleware [10], to accomplish the whole computation by splitting the analysis into 1.580 jobs, each consisting of 5.000 ESTs to be annotated against the modified UniProtKB database. In about four weeks all the results were collected, covering about 8 years of a standard computer activity, for a total of 6.946.250 hits in 577 MB of output files. The crunching factor, which basically represents the average number of CPUs used simultaneously along the computation, can be estimated to be roughly 100. This scalability is satisfactory considering the job submission rate, which was decreased due to the high quantity of data that had to be transferred within the grid infrastructure, both for uploading the UniProtKB database on the computational resources and to collect the output files.

**Figure 1:**
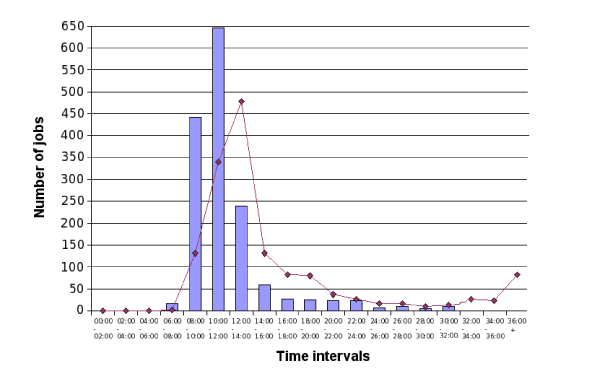
**Time statistics of the BLAST challenge**. The blue bars of the histogram represent the jobs rate of accomplishment in relation to the effective CPU time needed by the grid resources to perform the bare computation, while the red line represents the time needed to complete the job over the grid, from its submission to the complete retrieving of results.

From the computational point of view, the greatest problem of the EGEE grid is the dynamic behavior of the available resources. Due to network and system errors or in relation to the global computational load, the available resources are continuously reshaped, and the job rate of failure is quite high. Therefore, we employed a fault tolerant infrastructure to manage the whole computation procedure that, by tracing the status of the job constantly, immediately resubmits each task that presents problems or an inconsistent status [11]. Another issue to address was the usage of a large dataset of sequences over the grid, which we solved by replicating the UniProtKB database onto different grid storage facilities to reduce the data transfer time during the computation. At last, the reported performance (Fig. 1) includes the time spent to post-process the output files, a task that was accomplished directly on the grid computational resources to reduce the time required both for parsing the results and for uploading output records in the database.

### Ontology retrieval and establishment of relationships with GO categories and molecular pathways

A custom tool was prepared to collect and process annotation outputs from 10,000 randomly selected annotated sequences for each library. Sequences from a small number of under-represented libraries were also processed, as they belonged to biologically relevant tissues or health states. By means of the UniProt identifiers of the best BLAST hits, each EST was related to corresponding GO and pathway identifiers. In-house prepared scripts were used to create library-specific database tables containing the relationships between the proteins inferred from EST annotation and GO numbers. Based on these data, statistics are created and displayed at the web site. Statistical graphical displays are prepared on-the-fly by the web interface upon user's request, and are presented as proportional bars filling each class of the GO tree. The percentage of ESTs contributing to each category is given near the bars. The number of matching hits, together with the total number of sequences in the library, are also given near the bars, allowing the identification of under-represented libraries.

### Searching the Human EST Ontology Explorer database via the web interface

**Figure 2:**
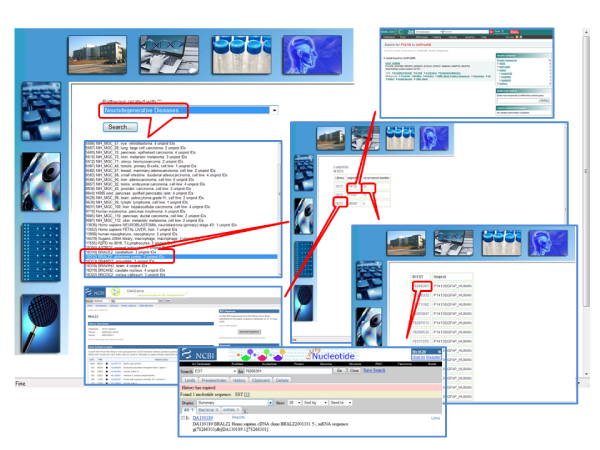
**The HEOE web pages: a pathway search example**. Examples of query output pages from a pathway search at the HEOE. The query started from pathway selection.

**Figure 3:**
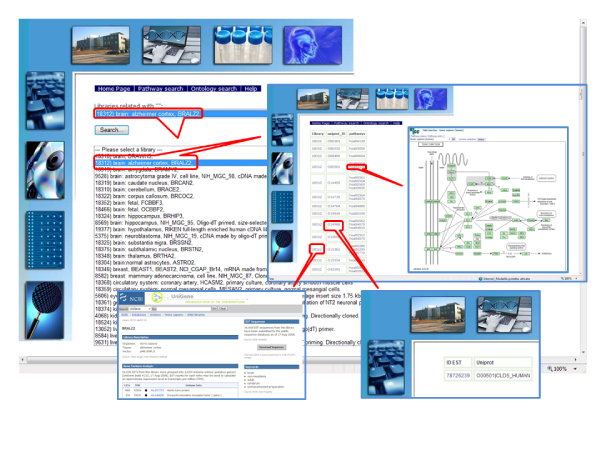
**The HEOE web pages: a pathway search example**. Examples of query output pages from a pathway search at the HEOE. The query started from library selection.

At the HEOE web site, pathway-oriented, ontology-oriented and statistics search pages are available. In the "Pathway search" page (Fig. 2 and Fig. 3), library involvement in metabolic pathways can be inspected, either focussing searches on selected pathways, or on users' libraries of interest. Two boxes are available to restrict searches to subsets of interest by text search on the description fields of the pathways or libraries. If a search is performed with blank "Pathway description" or "Library description" fields, a page is displayed giving a list of all the available pathways/libraries. Once a search is performed on a selected pathway, a list of all the libraries containing sequences related to that pathway is displayed, also reporting the number of matching hits. Clicking on a library, a list of the sequences involved in the pathway is given, together with links to the NCBI library details, to the corresponding UniProt entry page, and to the original NCBI dbEST sequences. When searches are performed on a selected library, all pathway-related sequences are shown for that library, with links to the NCBI library details, UniProt entry and KEGG pathway [12] pages.

**Figure 4:**
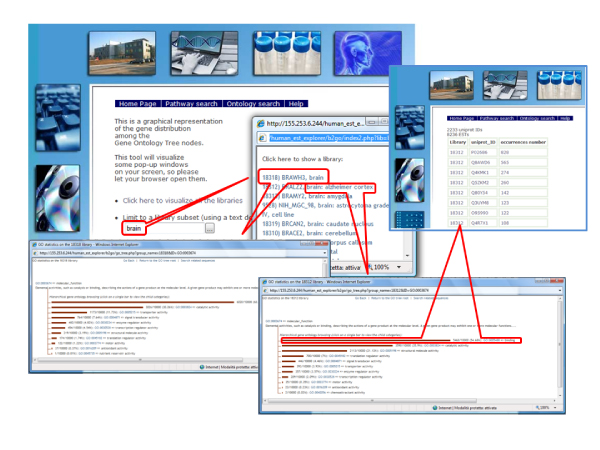
**The HEOE web pages: an ontology search example**. Examples of query output pages from an ontology search at the HEOE. Simultaneous display of GO statistics in different libraries allows direct comparison of the selected tissues.

Via the "Ontology search" page (Fig. 4) options, ontology statistics for different libraries can be simultaneously displayed in independent pop-up windows. A full list of libraries can be scrolled to select the libraries of interest, or a text search can be performed on the library description field to select subsets of libraries. In each library-dedicated window, ontological classes are displayed, each represented by a proportional bar. Bars can be clicked to move hierarchically across categories. When a bar is clicked, a page appears displaying the distribution of hits in category sub-classes, and a link is given at the top of the page to retrieve sequences that are included in the selected GO class. Retrieved sequences are presented in a table grouped by UniProt ID and single sequences related to each UniProt ID can be retrieved by clicking on the rightmost column value. As for pathway-oriented searches, all query outputs are linked to the NCBI library details, UniProt entry, and dbEST sequence pages.

In the "Statistics" page users can select a number of libraries for statistical comparison (from a minimum of two libraries to the whole library collection). Text searches on descriptions of libraries can be performed by filling the "Library description" box, to narrow the choice to specific groups of libraries. Following the selection of libraries, chi-squared values are computed for each GO category, and a table is displayed reporting the number of sequences in each library for each GO class, together with corresponding chi-squared values and P-values. Hits are sorted by descending chi-squared values. Clicking on the table nodes (i.e. on the number of ESTs of each library for each GO category), the corresponding proportional bar is displayed, allowing hierarchical browsing of the GO DAG and sequences retrieval. To speed up output reading and partially decrease information redundancy that is intrinsic to the GO DAG, statistics can be restricted to GO classes belonging to a generic GO slim [13] that represent the most significant classes of the GO DAG. In the statistics web page, users can choose to either retrieve full outputs, or retrieve only the GO slim related set of statistics.

## Discussion

The HEOE project was born with the dual intent to obtain comparable EST distributions in GO categories in different expression libraries and to test the grid computing scalability on large amounts of input data. To achieve this second goal, we decided to perform BLAST annotation on the entire human EST collection. Actually, this challenging task gave us the opportunity to test our computation system working on the top of the EGEE infrastructure, both optimizing the data processing procedures and tuning the post-processing analysis.

It should be noted here that in the activity we carried out during annotation of EST over the grid, about 63% of the jobs were reported as successfully finished, according to the status logged in the official grid monitoring system. However, the ratio decreased to 57% after checking for the existence of the output in the database. Nonetheless, failed jobs were resubmitted by the managing system until the analysis full accomplishment. The main cause of job failure seemed to be the data transfer between the computational resources during the database upload and, in a few cases, errors in the connection to the local MySQL database.

According to these data, we can argue that the fault tolerant system for managing grid computation employed in this work provides reliable advantages in managing large scale analysis, reducing the gap between requirements of the bioinformatics community and the potential of grid computing technology.

From the biological point of view, the HEOE offers an insight in tissue- or health state-specific gene expression levels that is complementary to that offered by other public resources devoted to digital differential display. None of the other resources, in fact, provides the possibility to perform library-driven pathway-oriented or ontology-oriented searches on the human transcriptome.

Comparing the HEOE to the HGI, a significant difference exists in dataset management. The main goal of the HGI database is not to provide a classical digital differential display system, but to give a non-redundant view of all human genes. Thus, at the HGI, ESTs are assembled together with transcript sequences to create Tentative Consensus sequences (TCs) that are subsequently annotated. When expression levels are compared between two libraries, participation of the libraries in TCs is displayed in terms of number of ESTs, where statistically significant differences are observable. In this way, no normalization is accomplished with respect to the number of sequences of each library, and no selection is performed on libraries according to their preparation method. Results from this analysis call for further investigation and validation, and users must acquire information about libraries for a proper interpretation of output. Classification of TCs in GO categories and their involvement in metabolic pathways are also provided at the HGI, but because these tools are applied to a non-redundant dataset, no quantitative evidence is given about the expression levels in different tissues or health states. Furthermore, because the TCs primarily comprise sequences from different libraries, it is very hard to investigate the presence of each library in different GO classes or pathways.

At the NCBI, the DDD tool allows highlighting statistically significant differences between user-defined pools of libraries in representation of ESTs. ESTs frequency values provide normalized information with respect to the total number of considered sequences, and details of libraries can be easily accessed to obtain information about preparation methods. This tool is conceptually similar to the HEOE, but no classification according to ontology categories or pathways is given.

A third tool for digital differential display is the ZooDDD [14], where expression differences between two species, tissues or developmental stages can be highlighted, and eventually displayed on the GO tree via the GOBU applet [15]. Nonetheless, the main goal of the ZooDDD is to mine evolutionarily conserved, highly expressed, tissue-specific orthologues in model animals, and selection of specific libraries is not allowed. Moving across GO categories via the HEOE graphical bars allows an immediate visual identification of differentially represented classes in the selected libraries. Consequent retrieval of GO class-related entries and of their expression level provides a means to individualize genes subject to variation in transcription in different tissues or health states. The possibility to perform statistics allows to readily identify significantly affected GO classes in subsets of libraries of interest. Furthermore, grouping transcripts according to ontologies allows one to focus on differentially expressed categories, and observe the general behavior of groups of genes, instead of considering entries independently. For example, comparing the "transcription factor activity" GO class (GO tree: molecular function (GO:000374), transcription regulator activity (GO:0030528), transcription factor activity (GO:0003700)) in two libraries from brain tissues – the former from normal tissue (library 18318) and the latter from Alzheimer cortex (library 18312) -, one can observe a different composition in the preferentially expressed proteins. This is a comparison that cannot be easily performed via other web tools.

## Conclusion

The HEOE allows browsing ontologies from 70 libraries from different tissues, but as we complete the evaluation of annotation results for the whole human dbEST dataset, inclusion of data from additional libraries is scheduled. The web interface offers the possibility to simultaneously visualize data from different libraries, allowing direct comparison of distribution of ontologies in user-selected tissues. Information retrieved via this tool can be used to complement and validate results obtained with alternative methods, contributing to deepen knowledge on gene expression differences among tissues and health states.

## Methods

### Libraries selection

The entire dbEST human dataset was downloaded from the NCBI, and a Perl script was prepared to group sequences according to the library-of-origin. 70 non-normalized libraries from healthy and unhealthy tissues (Additional file 1), most of which contained more than 10,000 sequences, were manually selected to create the initial dataset.

### EST annotation by BLAST

Six frame translations of the whole human dbEST collection were annotated by BLASTx against a custom-modified version of the UniProtKB database (a subset of SP-TrEMBL), where only sequences associated to GO terms were retained. In order to limit the whole computation time, grid computing technology was adopted, allowing the division of the computation in 1,580 jobs. BLAST results from sequences belonging to the selected libraries were parsed and stored in a MySQL database.

### Grid analysis managing system

The system employed to coordinate the grid computation is described in detail in Milanesi *et al *[11]. Briefly, it can be viewed as a double layered infrastructure that can be customized for different applications. The first layer is designed to control the execution of each single job and works directly on the top of the gLite middleware [10]. The second layer coordinates the distribution of the whole challenge by monitoring the Input/Output consistence of each job computed on the grid platform.

### GO Statistics and the web interface

Based on data contained in the Gene Ontology Annotation (GOA) Database [6] and in the Gene Ontology Database [5], Perl scripts were prepared to create a local database with all the protein-GO associations including no-direct links due to "is_a" relations among different GO elements. Information contained in the database tables was used to produce statistics on the distribution of ontologies. Graphical display and browsing of ontology classes is obtained via the PHP-based web interface, which produces graphical bars and matching ontologies percentages upon users' requests.

### Pathway-oriented EST classification

UniProt identifiers associated with annotated EST sequences were used to relate ESTs to the 345 molecular pathways that are described at the KEGG Pathway database [12]. UniProt-pathway inter-relationships were deduced from association files available at the KEGG ftp site.

### Chi-squared analysis on ontologies distribution in GO classes

Chi-squared computations are performed on the fly on user-selected libraries. Computations are based on matrices containing the number of ESTs for each GO element for all the libraries. To avoid discrepancies due to the presence of under-represented libraries, values in the matrices were normalized to a total number of 10,000 sequences for each library. Two different matrices were prepared: the former containing all the GO elements that are included in the GO DAG, and the latter containing only GO identifiers belonging to a generic GO slim [13], encompassing 132 GO entries.

## Competing interests

The authors declare that they have no competing interests.

## Authors' contributions

IM designed the infrastructure to perform the BLAST computation over the EGEE grid, accomplished the EST annotation against the UniProtKB database, and contributed to the manuscript preparation. AC structured the pipeline, the database, the GO statistics tool and the web interface, and wrote all the accessory programs. AS participated in the design of the study and critically revised the manuscript. MDC contributed to the implementation of the web interface. LM granted the access to the computational facilities and maintained the bioinformatics resources. BL defined the pipeline structure and the web interface contents and drafted the manuscript.

## Supplementary Material

Additional file 1 **The Human EST Ontology Explorer library collection**. A list of the libraries that are by now included in the HEOE. Library names and identifiers (ID) are as from the NCBI dbEST. Click here for file
