# Supplementary material for: The Human EST Ontology Explorer: a tissue-oriented visualization system for ontologies distribution in human EST collections
Source: BMC Bioinformatics. 2009 Oct 15;10(Suppl 12):S2. doi: 10.1186/1471-2105-10-S12-S2 (PMC2762067; doi:10.1186/1471-2105-10-S12-S2)
Supplement: Additional file 1 — The Human EST Ontology Explorer library collection. A list of the libraries that are by now included in the HEOE. Library names and identifiers (ID) are as from the NCBI dbEST. [file 1471-2105-10-S12-S2-S1.doc]

## Additional file 1. The Human EST Ontology Explorer library collection

A list of the libraries that are by now included in the HEOE. Library names and identifiers (ID) are as from the NCBI dbEST.

| **Tissue/Type** | **Library Name** | **Library ID** |
| --- | --- | --- |
| brain | BRAWH3 | 18318 |
| brain: alzheimer cortex | BRALZ2 | 18312 |
| brain: amygdala | BRAMY2 | 18313 |
| brain: astrocytoma grade IV, cell line | NIH_MGC_98 | 9528 |
| brain: caudate nucleus | BRCAN2 | 18319 |
| brain: cerebellum | BRACE2 | 18310 |
| brain: corpus callosum | BRCOC2 | 18322 |
| brain: fetal | FCBBF3 | 18352 |
| brain: fetal | OCBBF2 | 18466 |
| brain: hippocampus | BRHIP3 | 18324 |
| brain: hippocampus | NIH_MGC_95 | 8569 |
| brain: hypothalamus | RIKEN full-length enriched human cDNA library, hypothalamus | 19377 |
| brain: neuroblastoma | NIH_MGC_19 | 5375 |
| brain: substantia nigra | BRSSN2 | 18325 |
| brain: subthalamic nucleus | BRSTN2 | 18375 |
| brain: thalamus | BRTHA2 | 18348 |
| brain:normal astrocytes | ASTRO2 | 18304 |
| breast | BEAST1  BEAST2  NCI_CGAP_Br14 | 18346  18305  1445 |
| breast: mammary adenocarcinoma, cell line | NIH_MGC_87 | 8582 |
| circulatory system: coronary artery | HCASM2 | 18368 |
| circulatory system: normal mesangial cells | MESAN2 | 18359 |
| eye: retiniblastoma | NIH_MGC_67 | 5606 |
| germ cells: teratocarcinoma | NT2NE2 | 18361 |
| kidney | KIDNE2 | 18374 |
| kidney: renal cell adenocarcinoma | NIH_MGC_14 | 4068 |
| kidney: tumor tissue | TKIDN2 | 18524 |
| liver | Homo sapiens FETAL LIVER | 13052 |
| liver: adenocarcinoma, cell line | NIH_MGC_90 | 8584 |
| liver: hepatocellular carcinoma, cell line | NIH_MGC_100 | 9631 |
| liver: regeneration after partial hepatectomy | Human liver regeneration after partial hepatectomy | 18893 |
| lung | HLUNG2 | 18363 |
| lung: large cell carcinoma | NIH_MGC_68 | 5607 |
| lung: small cell carcinoma | NIH_MGC_7 | 2586 |
| lymph: Burkitt lymphoma | NIH_MGC_8 | 4069 |
| lymph: lymphoma, cell line | NIH_MGC_99 | 9630 |
| macrophage | Sugano cDNA library, macrophage | 16419 |
| muscle: rhabdomyosarcoma | NIH_MGC_17 | 3714 |
| nasopharynx | human nasopharynx | 13908 |
| nervous system (SNS): neuroblastoma | IMR322 | 18358 |
| nervous system (SNS): neuroblastoma (primary) stage 4S | Homo sapiens NEUROBLASTOMA | 13036 |
| ovary | Stratagene ovary (#937217) | 253 |
| ovary: adenocarcinoma cell line | NIH_MGC_9 | 4908 |
| pancreas | Sugano cDNA library, pancreas | 16423 |
| pancreas: ductal carcinoma, cell line | NIH_MGC_110 | 9885 |
| pancreas: epithelioid carcinoma | NIH_MGC_70 | 5609 |
| pancreas: insulinoma | Human insulinoma | 9716 |
| pancreas: purified pancreatic islet | HR85 islet | 8840 |
| placenta | PLACE6 | 18468 |
| placenta: choriocarcinoma | NIH_MGC_21 | 4070 |
| prostate | PROST2 | 18469 |
| prostate: carcinoma, cell line | NIH_MGC_40 | 8834 |
| skin: amelanotic melanoma, cell line | NIH_MGC_41 | 8775 |
| skin: melanotic melanoma | NIH_MGC_72 | 5610 |
| skin: melanotic melanoma, cell line | NIH_MGC_112 | 9901 |
| skin: neonatal normal dermal fibroblasts | DFNES2 | 18384 |
| small intestine | SMINT2 | 18473 |
| small intestine: duodenal adenocarcinoma, cell line | NIH_MGC_88 | 8583 |
| spleen | SPLEN2 | 18474 |
| synovial membrane tissue from rheumatioid arthritis | SYNOV2 | 18480 |
| testis | TESTI2 | 18476 |
| testis: embryonal carcinoma, cell line | NIH_MGC_92 | 8657 |
| thymus | THYMU3 | 18520 |
| T-Lymphocytes | RZPD no.9016 | 17555 |
| tongue: tumor tissue | CTONG2 | 18389 |
| tonsils: primary B-cells, cell line | NIH_MGC_48 | 6987 |
| trachea | TRACH3 | 18522 |
| uterus | UTERU2 | 18523 |
| uterus: cervix | NIH_MGC_10 | 2587 |
| uterus: cervix: cervical carcinoma cell line | NIH_MGC_12 | 4011 |
| uterus: leiomyosarcoma | NIH_MGC_71 | 5612 |
